# Neuronal Differentiation and Extensive Migration of Human Neural Precursor Cells following Co-Culture with Rat Auditory Brainstem Slices

**DOI:** 10.1371/journal.pone.0057301

**Published:** 2013-03-07

**Authors:** Ekaterina Novozhilova, Petri Olivius, Piyaporn Siratirakun, Cecilia Lundberg, Ulrica Englund-Johansson

**Affiliations:** 1 Department of ENT—Head and Neck Surgery, UHL, County Council of Östergötland, Linköping, Sweden; 2 Division of Oto-Rhino-Laryngology and Head and Neck Surgery, Department of Clinical and Experimental Medicine, Faculty of Health Sciences, Linköping University, Linköping, Sweden; 3 Center for Hearing and Communication Research, Karolinska University Hospital, Stockholm, Sweden; 4 Department of Clinical Neuroscience, Section of Otorhinolaryngology, Karolinska Institute, Karolinska University Hospital, Stockholm, Sweden; 5 CNS Gene Therapy Unit, Dept. of Experimental Medical Science, Lund University, Lund, Sweden; 6 Department of Ophthalmology, Institution of Clinical Sciences in Lund, Lund University, Lund, Sweden; University of South Florida, United States of America

## Abstract

Congenital or acquired hearing loss is often associated with a progressive degeneration of the auditory nerve (AN) in the inner ear. The AN is composed of processes and axons of the bipolar spiral ganglion neurons (SGN), forming the connection between the hair cells in the inner ear cochlea and the cochlear nuclei (CN) in the brainstem (BS). Therefore, replacement of SGNs for restoring the AN to improve hearing function in patients who receive a cochlear implantation or have severe AN malfunctions is an attractive idea. A human neural precursor cell (HNPC) is an appropriate donor cell to investigate, as it can be isolated and expanded *in vitro* with maintained potential to form neurons and glia. We recently developed a post-natal rodent *in vitro* auditory BS slice culture model including the CN and the central part of the AN for initial studies of candidate cells. Here we characterized the survival, distribution, phenotypic differentiation, and integration capacity of HNPCs into the auditory circuitry *in vitro*. HNPC aggregates (spheres) were deposited adjacent to or on top of the BS slices or as a monoculture (control). The results demonstrate that co-cultured HNPCs compared to monocultures (1) survive better, (2) distribute over a larger area, (3) to a larger extent and in a shorter time-frame form mature neuronal and glial phenotypes. HNPC showed the ability to extend neurites into host tissue. Our findings suggest that the HNPC-BS slice co-culture is appropriate for further investigations on the integration capacity of HNPCs into the auditory circuitry.

## Introduction

Severe hearing impairment is one of the most frequent disorders in society affecting millions of people in the industrialized world and even more in the developing countries, prevalence: ≥10% in Europe [1]. Since most types of congenital or acquired hearing loss to varying degree are associated with an irreversible loss of inner ear sensory hair cells and spiral ganglion neurons (SGN), replacement of SGN for regaining function is an attractive idea for future clinical therapies in selected groups of patients. The vast majority of hearing disabled people, predominantly having hair cell loss, can regain good hearing function by using conventional hearing aids. In severe cases, however, the hair cells and/or the SGN have degenerated to such an extent that the conventional hearing aid can no longer provide the assistance needed for good hearing even in unchallenging hearing environments such as a two person conversation in a closed room. In here and similar cases a cochlear implant (CI) directly stimulating on the auditory neurons, thereby bypassing the hair cells, is usually needed [2]. However, even though a CI may halt the neuronal degeneration process in the auditory circuitry it will function significantly less well or not at all if the SGN have already degenerated.

Extensive research over the last two decades demonstrates that isolated mammalian, including human, embryonic or adult stem- and progenitor cells retain the potential to form neurons and glial cells after *in vitro* expansion [3], [4]. Therefore, such cells represent an interesting option as donor material for cell replacement in various degenerative diseases and could theoretically serve as a cell bank for a clinical use [5]–[9]. Indeed, numerous reports using stem- and progenitor cells in a wide range of neurodegenerative disease models describe good survival, region-specific neuronal differentiation as well as functional recovery [10]–[12].

Since the auditory system like the majority of regions of the central nervous system (CNS), has a restricted regenerative potential [13], stem cell transplantation has been proposed as an option for treating auditory degenerative disorders. More than a decade of intensive pre-clinical studies evaluating potential stem cell types, ranging from embryonic stem cells (ESCs) to inner ear progenitor cells, has proven that both hair cells and SGN can to some extent be replaced [14]–[32]. Encouragingly, even functional recovery after grafting of adult human olfactory stem cells was demonstrated in a model of sensory-neural hearing loss [32]. In agreement, in several reports our laboratory describes good survival, neuronal differentiation and to some extent donor-host integration after *in vivo* transplantation of e.g. mouse ESCs to the adult inner ear [33]–[38]. Recently, our laboratory successfully established and efficiently used a rodent organotypic tissue slice model of the auditory brainstem (BS) for initial validation of potential donor stem cells [39]–[42]. The present *in vitro* model contains part of the auditory BS neural circuitry, including the cochlear nucleus (CN, i.e. the target neurons of the SGN) and a minor part of the auditory nerve (AN). The BS slices in our model maintain their three-dimensional organization for up to five weeks in culture, and, thus serve as a controlled organotypic system where various experimental approaches for AN reconstruction can be evaluated, including pharmacological treatments and a cellular SGN replacement therapy [42]. We have reported that mouse ESCs survive well and have an increased neuronal differentiation when co-cultured with the BS slice as compared to in monoculture [40], [41]

Here we investigate whether also human neural stem cells have the ability to respond to the permissive environment provided by the BS culture for survival and neuronal differentiation. Furthermore, the potential of the human cells to migrate into and extend neurites directed toward the CN was examined. We speculate that the use of donor cells of human origin may be an important step towards a future clinical setting, where implantation of similar cells will most likely be required. We employ a fetal human neural cell line that can be stable long-term mitogen-expanded *in vitro*, retain multipotency *in vitro* as well as *in vivo* after experimental grafting to the neonatal and adult rodent brain [43]–[45]. The cell line was established from the forebrain of a fetal brain, with no cloning and is therefore comprised of immature neural cells ranging from neural stem cells to early neural progenitors [45]. As such, we hereafter define the cells as human neural precursor cells (HNPCs). In this paper, we demonstrate that the HNPCs have the capacity to survive, migrate, form neurons and to some extent integrate with host tissue after four weeks of co-culture with a rat BS slice. Monocultured HNPCs served as controls. Significantly better survival, increased migration and neuronal differentiation of the HNPCs were shown after co-culture as compared to monoculture. Therefore, we have selected the presently used HNPCs as a most promising candidate for further investigations on how the integration capacity can be improved using the present co-culture assay as well as for *in vivo* transplantation in appropriate models of sensory-neural hearing loss.

## Materials and Methods

### Generation and expansion of the human neural precursor cell line

The human neural precursor cell line used for this study was originally established by L. Wahlberg, Å. Seiger, and colleagues at the Karolinska University Hospital (original work with the cell line is described in [45] and was kindly provided to us via Prof. A. Björklund (Dept. Exp. Med. Sci., Lund University, Sweden). Briefly, forebrain tissue was obtained from one 9-week (post conception) human embryo. The HNPC cell line derived from the embryo was maintained as free floating clusters (neurospheres) in defined DMEM-F12 medium supplemented with 2.0 mM L-glutamine (Sigma), 0.6% glucose (Sigma), N2 supplement (Invitrogen) and 2.0 µg/ml heparin (Sigma). The growth factors human basic FGF (hbFGF, 20 ng/ml; Invitrogen), human EGF (hEGF, 20 ng/ml; R&D Systems) and human LIF (hLIF, 20 ng/ml; Sigma) were added every third day to the culture. The neurospheres were passaged by mechanical dissociation every 7–10 days and reseeded as single cells at a density of 1×10^5^ cells/ml. Cells used in this study had been passaged three times before transplantation. A fraction of the HNPC expressed the reporter gene green fluorescent protein (GFP), transduced to the cells using a lentiviral infection. Multiplicity of infection (MOI) of 0.1 was used to infect the HNPC rendering 10% of the cells GFP positive prior to seeding. For details on lentiviral infection see [46].

### Animals

Postnatal Sprague-Dawley (SD) rat pups (P12–P14, n = 10) used for organotypic BS slice culture were obtained from Harlan (the Netherlands). The animals were maintained under standard conditions with food and water available *ad libitum*. All animal procedures were conducted in accordance with local ethical guidelines and approved animal care protocol (approval N329/07).

### Organotypic brainstem slice cultures

The organotypic BS slice culture protocol has been described previously [42]–[43]. Briefly, SD rat postnatal pups (P12–P14) were sacrificed by decapitation after pentobarbital sodium overdose. Then skulls were opened longitudinally along the midline and excised brains were placed in the ice cold dissecting medium (Hank's balanced salt solution (HBSS) supplemented with 20% glucose and 1% antibiotic-antimycotic (Penicillin/Streptomycin/Amphotericin B; Invitrogen). Three hundred micrometer thick transverse sections of the brainstem encompassing the proximal part of the cochlear nerve and the CN according to local anatomical landmark were obtained using a tissue-chopping device (McIlwain). The BS slices were transferred to petri dishes and kept in dissecting medium until further separation of the individual slices. Slices were propagated as interface cultures (37°C, 5% CO_2_) on polyester membranes with 0.4 µm pore size and 24 mm in diameter (Corning Inc), coated with poly-D-lysine hydrobromide (10 µg/ml; Sigma) and laminin (10 µg/ml; Invitrogen). Culture medium consisted of DMEM (high glucose, L-glutamine, pyruvate; Gibco) supplemented with 30% HBSS, 10% FBS, glucose (6.5 g/l), HEPES (25 mM) and 1% antibiotic/antimycotic. The culture medium was changed the day after preparation of the slices followed by every other day medium change routine during the culture period. Slices with abundant gliosis or visible neuronal loss were discarded.

### Experimental groups: monocultures and co-cultures

To observe whether the HNPC-spheres could survive and differentiate in co-culture with rat auditory BS slices, spheres 0.3 mm or 1 mm in size were deposited at a 0.5 mm distance next to or on top of the CN of the BS slice and the transected vestibulocochlear nerve. The deposition was conducted four days after start of the BS slice culture. The medium in the co-cultures was changed every other day. During the entire culture period a Nikon inverted microscope TS100 (Nikon, Kanagawa, Japan) was used to monitor cell morphology, survival and distribution. Following two and four weeks after HNPC deposition the experiments were terminated by fixation of the cultures (see below). See Table 1 for a summary of the experimental groups.

**Table 1:** Experimental groups, including size of seeded spheres, sphere placement with regard to the BS slice, survival times and rates.

Sphere size (mm)/placement	Survival time (weeks (w))	Number of surviving HNPC
*Monocultures*		
0.3	2 w	3/6 (50%)
1.0	2 w	9/18 (50%)
0.3	4 w	2/9 (22%)
1.0	4 w	7/12 (58%)
*Co-cultures - adjacent*		
0.3 mm	2 w	8/8 (100%)
1.0 mm	2 w	6/6 (100%)
0.3 mm	4 w	6/6 (100%)
1.0 mm	4 w	6/6 (100%)
*Co-cultures – on-top*		
1.0 mm	2 w	2/2 (100%)
1.0 mm	4 w	4/4 (100%)

The survival rate was measured as number of specimens displaying ≥40 human nuclei-positive cells/the total numbers of spheres seeded in the respective group.

### Immunohistochemistry

All cultures, i.e. mono- and co-cultures, were fixed in 4% paraformaldehyde (PFA) in phosphate- buffered saline (PBS) for 1 h at room temperature (RT) and then incubated in ice-cold 20% methanol in PBS for 5 min at RT. Specimen permeabilization prior to all immunostaining was done by incubation in 0.5% Triton X-100 in PBS overnight at 4°C. Before adding the primary antibody all specimens were pre-treated for 12 h at 4°C with 20% BSA in PBS blocking solution. Cultures were then incubated with different primary antibodies diluted in 5% BSA in PBS overnight at 4°C. The following primary antibodies and dilutions were used: β-tubulin III (Tuj1, 1∶200, Covance), Doublecortin (DCX, 1∶200, Abcam), glial fibrillary acidic protein (GFAP, 1∶500, DakoCyto, Denmark A/S), green fluorescent protein-FITC (GFP-FITC, 1∶200, Abcam), human specific nestin (1∶500, Chemicon International), human specific nuclei (h nuc, 1∶200, Millipore (Chemicon)), human Tau (hTau, 1∶200, Santa Cruz Biotech., Inc), synaptic vesicle protein (SV2A, 1∶200, Santa Cruz Biotechnology). Thereafter, appropriate fluorescent secondary antibodies were applied (all obtained from Jackson Immuno Research, Inc) for 4 h at RT followed by incubation with 20 µg/ml 4′,6-diamidino-2-phenylindole (DAPI) for 10 min. Cultures grown on membranes were cut out and mounted on glass slides with Prolong Gold mounting medium (Invitrogen).

### Analysis using fluorescence and confocal microscopy

Analysis on survival rate of spheres, cell distribution area and phenotypic differentiation of the human cells was performed using fluorescence and confocal microscopy (Axio Observer Z1 and LSM700, Zeiss) and ZEN2010 imaging software (Carl Zeiss MicroImaging GmbH, release version 6.0).

#### Survival rate

The survival rate at two and four weeks of the seeded spheres were calculated as number of specimens with detectable HNPC divided by the total number of spheres seeded in the respective group expressed as percentage. The threshold for a surviving sphere was set to detection of ≥40 cells. Results are given as percentage. Human cells were detected by immunohistochemical staining with a human-specific nuclear marker and/or GFP-expression. It was out of the scope of the present study to determine the exact number of surviving human cells.

#### Distribution area

The total area over which the HNPC distributed after mono- or co-culture was measured at two and four weeks. All specimens with surviving HNPC (see above) were included in this analysis. Here human nuclei-immunostained specimens were used since about only 10% of the HNPC are assessed to be expressing GFP, and could thus give an underestimation of the distribution area. ImageJ GRID plugin was used to grid low magnification (5×) images of the total HNPC distribution area. The grid square size was automatically set at 50 µm by 50 µm with an inclusion threshold of at least 50% coverage by the cell mass. Results are presented as mean area covered in mm^2^ ± SD, for the respective experimental group. The Mann-Whitney non-parametric test was used for the statistical analysis, with *p* values less than 0.05 considered statistically different.

#### Quantification of phenotypic differentiation

Cell counting of cells expression certain markers was done on real-time images acquired using 100× acquisition frame on LSM 700 laser scanning microscope (Zeiss) and ZEN 2010 imaging software (Carl Zeiss MicroImaging GmbH; release version 6.0). For each specimen, 30–50 randomly selected frames were acquired and the numbers of DAPI-labeled nuclei and cells with a respective phenotypic marker were counted per frame. Total numbers of cells expressing immature (nestin), glial (GFAP) and neuronal markers (Tuj1, DCX and hTau) were counted per frame and the data presented as a percentage of the total DAPI cell count. Specimens rendering a total cell count of ≥40 cells were included for further statistical analysis. All results are presented as mean ± SD and statistical analysis was performed between mono- and co-cultures as well as between the different time-points within the same group. The Mann-Whitney non-parametric test was used for the statistical analysis, with *p* values less than 0.05 considered statistically different.

## Results

After HNPC monoculture or co-culture with the BS slice; survival, distribution, phenotypic differentiation as well as extent of integration with the host tissue were examined. Analysis was made after either 2 or 4 weeks of culturing. The 2 weeks time-point was chosen for comparison to our recent study with mouse ESCs as donor cells [41]. The 4 weeks survival time was included as it has been previously shown that human neurons mature slower in culture as compared to their rodent counterparts and for comparison to *in vivo* phenotypic differentiation of similar grafted cells [43], [44].

In some of the co-cultures, the spheres were placed at a 0.5 mm distance from the BS slice border adjacent to the axotomized AN. The placement of the HNPCs donor cells in close vicinity of the AN was selected in order to illustrate the potential integration of the donor cells with the second order auditory neurons in the CN.

Prior to deposition of the spheres the fraction of HNPCs expressing GFP was estimated to be approximately 10% (lentiviral infection with a MOI of 0.1). Already at 2 weeks after co-culturing a decrease in the fraction of GFP-expressing cells was noted, suggesting a down-regulation of the reporter gene due to phenotypic differentiation [43], [44], [46] or possibly due to a more permissive environment for proliferation of non-GFP-expressing cells, which was not addressed in the present study.

### HNPCs in monoculture

#### Survival and migration

See Table 1 for a summary of experimental groups, including number of specimens, survival times and survival rates. At 2 weeks 50% of the monocultured specimens had survived, independently of sphere size used. However, in the 4-week monocultures, specimens originating from larger spheres illustrated better survival (58%) than the corresponding HNPCs from small spheres (22%).

**Figure 1:**
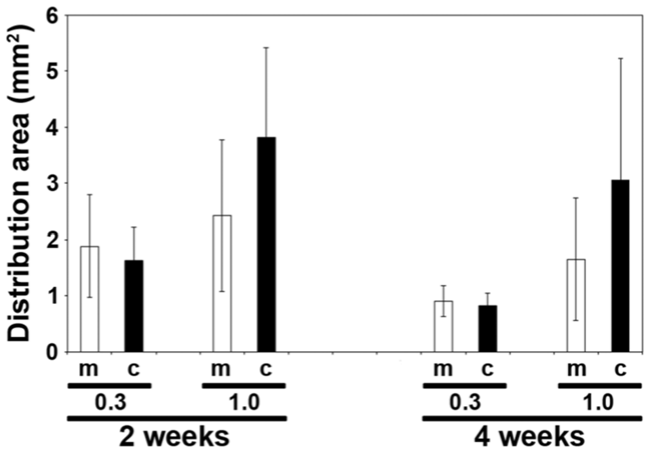
Distribution area of HNPCs after monoculture or co-culture. The graph shows the mean distribution area in mm^2^ for HNPCs after monoculture of co-culture after adjacent placement to an organoypic cultured brainstem slice. The area was estimated for all groups including 0.3 mm and 1.0 mm sized spheres, at 2 or 4 weeks of culture. All samples with surviving cells were included in the measurements (n = 2–9). Bars represent mean±SD, and white bars represent monocultures and black bars co-cultures. m = monoculture; c = co-culture.

The mean distribution area of the HNPCs for the respective groups can be found in Figure 1. There was a large variation in the size of the area found in all monoculture groups, which could be explained by the difficulties in estimating the size of the seeded spheres. The area was smaller at the longer survival time for the respective size group of spheres used, e.g. for 0.3 mm spheres the mean area was 1.9±0.9 mm^2^ at 2 weeks and 1.0±0.3 mm^2^ at 4 weeks. In parallel, for 1.0 mm spheres the area was 2.4±1.3 mm^2^ (2 weeks) compared to 1.7±1.1 mm^2^ (4 weeks). No obvious difference in the area covered by HNPCs was noted between mono- and co-culture after seeding of 0.3 mm sized spheres. However, for co-cultured 1.0 mm spheres the distribution area was larger than the monocultures both at 2 weeks (co-culture: 3.8±1.6 mm^2^) and 4 weeks (co-culture: 3.1±2.2 mm^2^). However, no attempt was made to quantify the exact numbers of human cells in the mono- and co-cultures. A common property of all groups was that the distribution area did not increase from 2 weeks to 4 weeks of culture.

**Figure 2:**
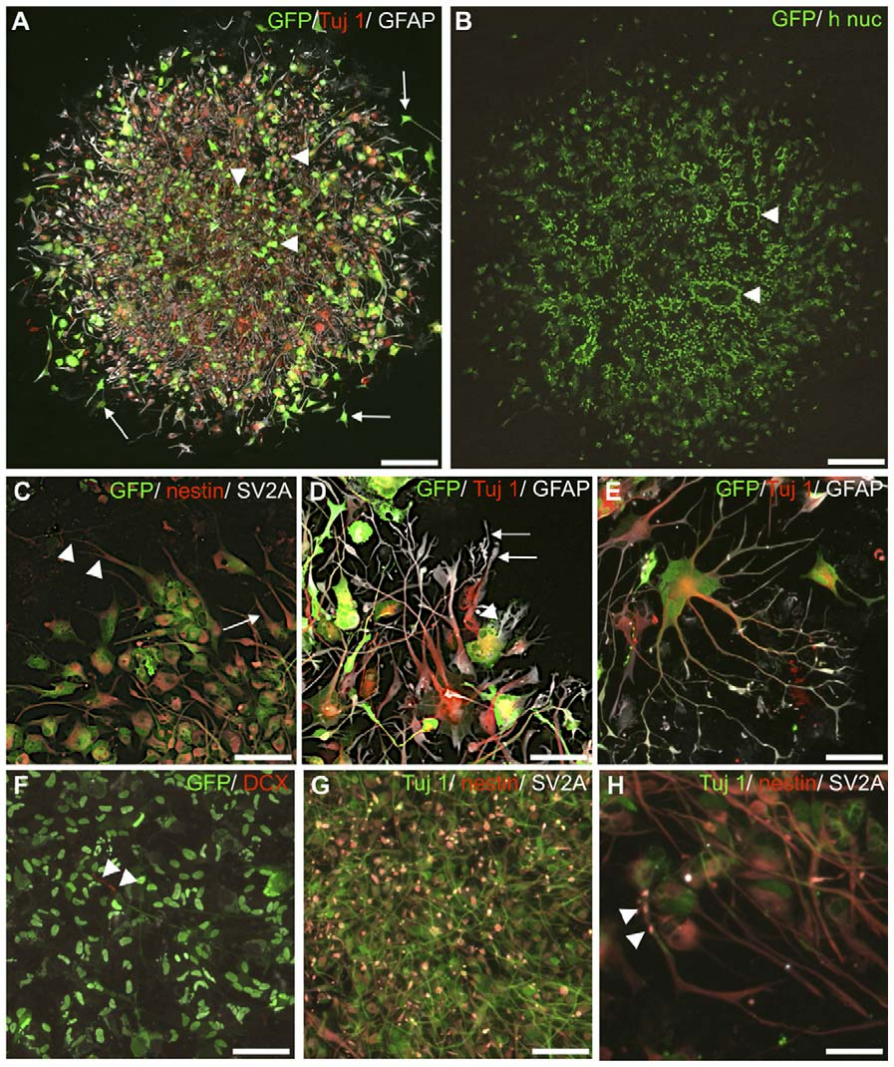
Distribution and differentiation of monocultured HNPCs. **A**. Up to 4 weeks the sphere remained intact with only limited migration observed. At both time-points GFP-expressing cells (green) expressed mainly immature cell profiles (arrowheads). At the periphery of the sphere bi- or multipolar GFP+ morphologies was located (arrows), expressing neuronal- (Tuj1, red)- and glial (GFAP, white) markers. **B**. Human nuclei- staining (h nuc, green) revealed a similar distribution pattern as the GFP-staining in A. Rosette-like cell clusters was noted (arrowheads). **C**. Neural progenitor marker nestin (red)-expression was abundant. These cells displayed apical (arrow) or bifurcated (arrowheads) leading processes and elongated cell bodies. **D**. In the periphery many multi- branched processed (arrows) cells co-expressed GFAP (white) and Tuj1 (red, arrowhead)+ cell bodies, suggesting on-going differentiation. **E**. A typical GFAP (white)/Tuj1 (red)+ human cell, with complex extensions. **F**. DCX (red)+ maturing neurons (arrowheads). **G**. SV2A (white) was expressed in a large fraction of HNPCs in the center. **H**. SV2A (arrowheads) co-localized with Tuj1 (green)+ process. Scale bar equals 75 µm in A, B; 100 µm in C–F; 50 µm in H.

Overall, at 2 weeks after deposition all of the spheres had attached and formed a monolayer of cells and the majority of the human nuclei- and GFP+ cells were evenly distributed in a circular pattern surrounding the core of the sphere at 4 weeks (Figs. 2A, B). Three of the four monoculture groups (i.e. excluding 0.3 mm spheres at 2 weeks), displayed many rosette formations restricted to the remaining center of the sphere (Fig. 2B). At both 2 and 4 weeks HNPCs displaying morphologies of migratory cells were detected, with typical elongated cell bodies and apical leading processes expressing nestin or Tuj1 (Fig. 2C). Taken together, moderate survival and limited cellular distribution was found after monoculture of HNPCs.

#### Neuronal and glial differentiation

After 4 weeks of monoculture 93.1±4.4% of the HNPCs still expressed the neural progenitor marker nestin, confirming a maintained undifferentiated stage for the majority of the HNPCs ([47]; Table 2). At 2 weeks, 97.0% (n = 2) of HNPCs were nestin+. These cells were situated in the center of the sphere as well as scattered in the outer region of the distribution pattern (Fig. 2C). Located in the center of the cell mass nestin+ HNPCs possessed immature cell morphologies with rounded cell bodies and few short processes (Fig. 2C). In some cases cells co-expressed the neural progenitor/astrocyte marker glial acidic fibrillary protein (GFAP) with the neuronal marker Tuj1, indicating on-going phenotypic differentiation (Figs. 2D, E). GFAP was previously described as a marker for immature neurons in similar cell lines [48] as well as for neuronal progenitors in neurogenic regions in the CNS [49]. Here the percentage of GFAP+ cells remained similar from 2 to 4 weeks in culture, i.e. 77.5±19.9% and 73.2% (n = 1) (Table 2). At both time-points the GFAP+ progenitors displayed similar morphologies as described above for the immature nestin+ cells. Few scattered mature glial cells in culture exhibited large flattened cell bodies (Fig. 2A).

**Table 2:** Expression of immature markers and glial as well as neuronal markers in HNPC in monoculture and in co-culture with auditory brainstem slices.

Survival time/Marker	Monoculture (% (n))	Co-culture (% (n))
*2 weeks*		
Nestin-positive	97.0 (2)	80.2±2.9 (3)
GFAP-positive	77.5±19.9 (5)	45.5±10.7 (6)
Tuj1-positive	77.5±17.3 (5)	49.8±3.3 (3)*
DCX-positive	98.6±2.0 (3)	79.1±6.9 (3)
hTau-positive	52.3±31.3 (3)	87.8 (2)
*4 weeks*		
Nestin-positive	93.1 (2)	-
GFAP-positive	73.2 (1)	56.7±8.7 (4)
Tuj1-positive	75.2 (1)	67.1±9.7 (4)
DCX-positive	100±0.0 (3)	74.2±8.4 (4)*
hTau-positive	90.7±4.6 (3)	46.3±12.4 (4)*

Numbers of HNPC expressing the respective markers above were estimated in monocultures and in cultures with an auditory BS slice, at 2 and 4 weeks after plating. The relative proportion of HNPC expressing the respective marker is expressed as mean±SD. Statistical difference between monoculture and co-culture as well as between time-points within the same groups was estimated using the Mann-Whitney nonparametric test. Statistical differences were only found between experimental groups,

* p<0.05. n = numbers of specimens.

At 2 weeks Tuj1 was expressed in 77.5±7.3% of the HNPCs, and a similar number (75.3%, n = 1) was estimated at 4 weeks (Table 2). Only in monocultures sprouting of the apical parts of the Tuj1+ processes was observed (Figs. 2D, E). DCX, expressed in migrating neuroblasts up to 4 weeks of differentiation [50] was expressed by 98.6±2.0% at 2 weeks and by all HNPCs (Fig. 2F; Table 2). Here, DCX was confined to the cell nucleus of all cells. In 2 and 4 week monocultures, hTau, a microtubule-associated protein [51] was expressed in 52.3±31.3% and in 90.7±4.6% of the HNPCs, respectively (Table 2). Although the numbers of hTau+ cells increased over time this finding was not accompanied by a change in neuronal morphologies toward a more complex neuronal profiles. Staining with the marker SV2A, which labels presynaptic vesicles, generated a diffuse staining localized to the cell bodies throughout the specimen (Fig. 2G, [52]). Although, SV2A expression could be co-localized in some Tuj1 positive processes, it is difficult to draw any conclusion of the staining since it renders a possible unspecific staining. (Figs. 2G, H).

In summary, the majority of monocultured HNPCs express nestin for up to 4 weeks and only occasional mature glial- and neuronal morphologies were found. However, in line with previous studies initial intrinsic guided glial- and neuronal formation was confirmed based on HNPC expression of specific markers for these cell types [45].

### HNPCs in co-culture

#### HNPCs adjacent to auditory BS slices—survival and migration

**Figure 3:**
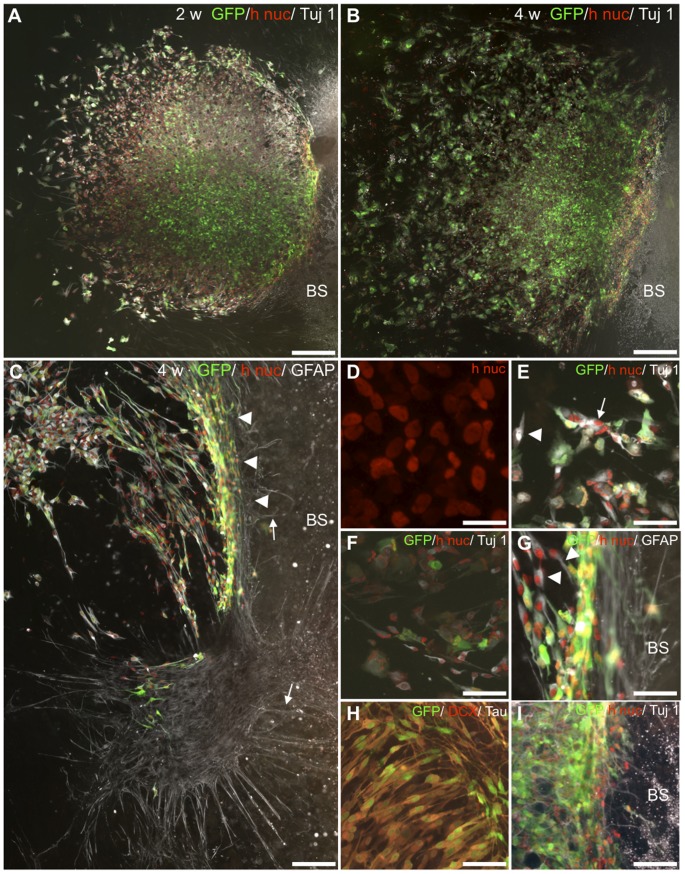
Distribution and migration of HNPCs cultured adjacent to BS slices. **A**. At 2 weeks migration of h nuc (red) and GFP (green)+ HNPCs was found directed both opposite and towards the BS. Note the large fraction of Tuj1 (white)+ HNPCs located at the sphere periphery. **B**. At 4 weeks the distribution of h nuc (red)/GFP (green) positive cells was more extensive cf to 2 weeks, with densely packed HNPCs found at the graft/host border. **C**. A stream of migrating h nuc/GFP/GFAP+ cells toward the host was seen. Only at 4 weeks a visible rim (arrowheads) at the donor-host border of cells was found, including very extensive GFAP+ processes (arrows) **D**. At the sphere core at both time-points densely packed HNPCs nuclei were found (h nuc, red). **E**. Tuj1 (white)/h nuc (red)-positive cells exhibited immature (arrow) and migrating cell (arrowhead) profiles in the periphery. **F**. H nuc (red)/Tuj1 (white)+ migrating cells were found at the periphery (arrowheads). **G**. H nuc (red)/GFAP (white)- expressing migrating HNPCs (arrowheads) were observed with an increasing toward the host. **H**. GFP/DCX (red)/hTau (white)+ cells were found migrating in parallel in a chain-like formation perpendicular to the BS slice border. **I**. Neuronal HNPCs, h nuc (red)/Tuj1 (white)+, found within the host tissue. BS = brain stem slice. Scale bar equals 200 µm in A, B; 100 µm in C, 25 µm in D, 50 µm in E–H, 100 µm in I.

All BS slice cultures survived well for up to 4 weeks and, in accordance with recent reports from our laboratory, kept their three-dimensional structure as judged by their morphology [40], [41]. A one hundred percent survival ratio was found for all co-cultured spheres (32/32, Table 1), judged by the detection of human nuclei (h nuc)- positive cells. In spheres of both sizes plated adjacent to the BS, large numbers of HNPCs survived well, as demonstrated by h nuc-and GFP-expression staining (Figs. 3A–C).

**Figure 4:**
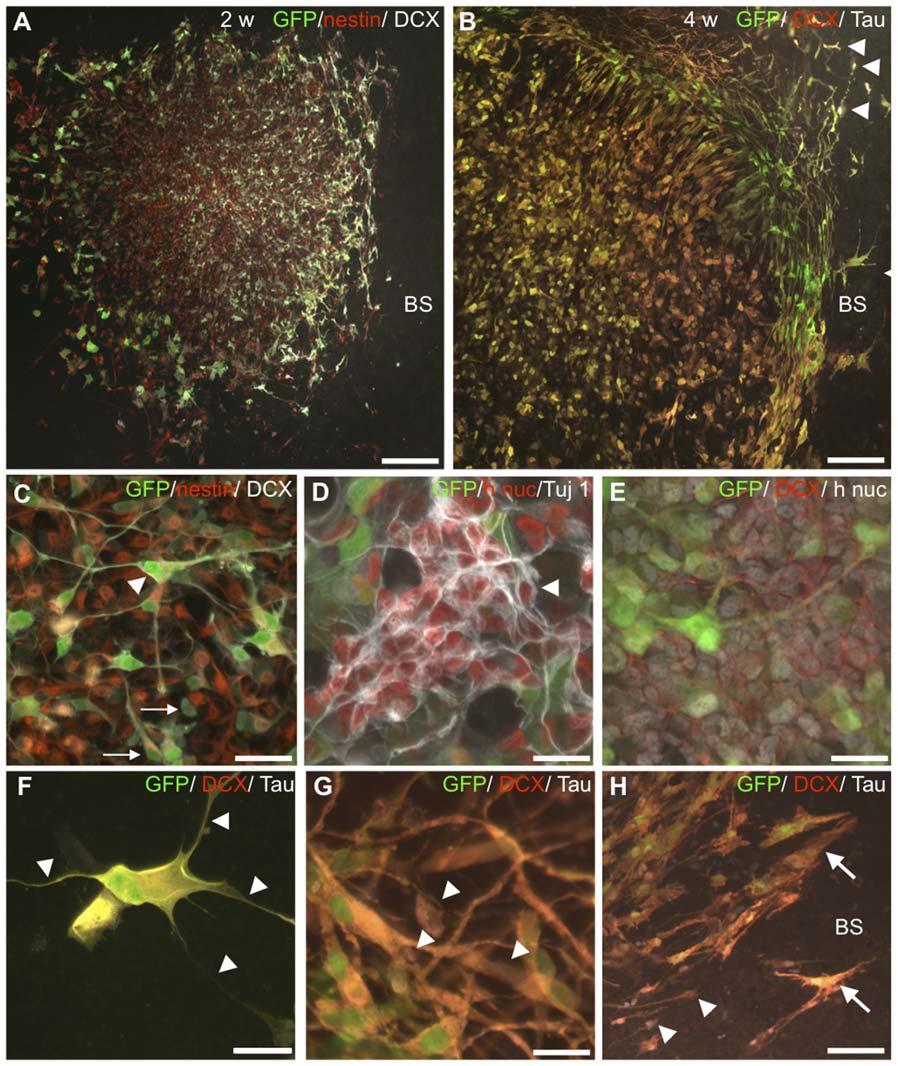
Increased neuronal differentiation in adjacent co-cultured HNPCs. **A**. DCX-staining (white) revealed an obvious preference of neuronal differentiation of HNPCs closer to the BS slice. In agreement, a significant decrease in numbers of nestin-expressing HNPC occurred from 2 to 4 weeks of co-culture. **B**. At 4 weeks a pronounced neuronal differentiation had occurred and many GFP/DCX (red)/hTau (white) double-labelled HNPCs were found. **C**. At 2 weeks a significant number of HNPCs were found in a transitional differentiation stage judged by the co-expression of nestin (red) and DCX (white, arrows). But, here also DCX+ cells with neuron-like profiles were found (arrowhead). **D**. Several “neurogenic” clusters of approximately 10–30 h nuc (red)/Tuj1 (white) were found at 2 weeks (arrowhead). **E**. Similar clusters expressing DCX (red) were also identified. **F**. At 4 weeks a large number of DCX (red)/hTau (white)+ HNPCs displayed complex morphologies with multi-branched extensions (arrowheads). **G**. GFP (green)/DCX (red)/Tau (white)+ HNPCs observed close to the BS tissue at 4 weeks. These cells most often exhibited profiles of migrating cells or early/late differentiated neurons (arrowheads). **H**. Near and within the BS slice GFP (green)/DCX (red)/Tau (white)+ HNPCs were both migrating (arrowheads) and maturing (arrows). Scale bar equals 200 µm in A; 100 µm in B; 50 µm in C–F, 25 µm in F, G and 40 µm in H.

Three main features characterized the distribution of human cells after the co-culturing. At first a remaining core of densely packed cells were found at both time-points (Figs. 3A–C). Notably, in the sphere core h nuc+ cells displayed rounded nuclei suggesting them being in non-migratory stage (Fig. 3D). Secondly, undirected continuous distribution of cells occurred in the opposite direction from the BS slice from 2 (Fig. 3A) to 4 weeks (Fig. 3B). In the periphery opposite from the BS slice, HNPCs displayed morphologies of both migrating and differentiating cells (Figs. 3E, F). Thirdly, directed migration and an increasing density of h nuc- and GFP+ cells toward- and to some extent into the BS slice was observed (Figs. 3B, C). Typically, densely packed GFAP- (Fig. 3G), DCX- (Fig. 3H) and Tuj1- (Figs. 3B, I)- expressing GFP+ human cells displaying morphologies of migrating cells oriented towards the BS were found. In some cases such cells formed chains of migrating cells, comparable to cell migration reported in the rostral migratory stream and in the developing cortex (Fig. 3H; and cf to ref [53]).

Human cells had the capacity to enter the host tissue, exemplified by a number of h nuc/GFP+ cells found in the outer region of the BS slice (Figs. 3G–I). Such cells expressed especially the neuronal markers DCX (Fig. 3H) and Tuj1 (Fig. 3I). Notably, in some specimens at the border between the host and transplanted cells a visible rim of a large number of HNPCs migrating along the outer host border was detected (Fig. 3C). The rim of HNPCs was more pronounced at 4 weeks of co-culture as compared to the earlier time-point studied. HNPCs found at the rim expressed both glial (GFAP-stained; Figs. 3C, G) and neuronal markers (β-tubulin III/DCX; Figs. 3I, H) and extended processes into the host tissue. In the current study, the limitation of a marker specific for human cells independent of stage of differentiation made it difficult to estimate the actual potential of the human cells to enter the host tissue, since the markers used here, GFP and human nuclei marker, are known to some extent to be down-regulated as the cells differentiate [43], [44], [46], [54], [55].

Taken together, co-cultured HNPCs show excellent survival and extensive directed migration toward the BS slice, suggesting that the human cells can i) respond to survival- and migration promoting cues exerted from the rat tissue or ii) are driven by repellant cues from neighbouring HNPCs for migration.

#### HNPCs adjacent to auditory BS slices—neuronal differentiation

After 2 weeks co-culture 20% less HNPCs expressed nestin as compared to monocultures at 2 weeks (80.2±2.9% and 97.0±0.9%, respectively; Table 2). At both survival times, immature nestin+ morphologies, with flat cell bodies and short unbranched processes were found as in the monocultures (Figs. 3A, C). On the other hand, in co-cultures nestin-positive cells exhibiting maturing/migrating morphologies, with slender cell bodies as well as extensive- and often multi-branched processes were found (Fig. 3C). In agreement, several “neurogenic” clusters of approximately 10–30 HNPCs with immature cellular profiles expressing Tuj1 and DCX were found at 2 weeks (Figs. 3C–E). In these clusters no GFP-expression was found. This is in agreement with previous studies indicating down-regulation of GFP in some cells upon differentiation [43], [44], [46], [54], [55]. Such neurogenic clusters were neither detected in the corresponding monocultures nor in co-cultures at 4 weeks, together indicating a continuous maturation/migration of the HNPCs into a neuronal lineage. This was further proven by the increase in number of Tuj1+ cells between 2 and 4 weeks of co-culture, cf. 49.8±3.3% to 67.1±9.7% (Table 2). For corresponding monocultures the fraction of Tuj1+ cells did not increase between time-points (77.5±17.3%; cf. to 75.3% (due to poor cell survival of 4 week monocultures only one sample was included)), see Table 2.

Also morphological analysis confirmed the progressing neuronal formation from 2 to 4 weeks for co-cultured HNPCs. This can be exemplified by the fact that no mature neuronal profiles could be detected in the monocultures. Already at 2 weeks, Tuj1+ human cells expressed morphologies of migrating or early/late differentiating neurons (Figs. 3A, E, H). DCX-expressing cells confirmed the on-going neurogenesis (Fig. 4C). A gradient of increasing numbers of DCX+ cells facing the BS slice was observed, suggesting that the BS slice may exert signals for neuronal migration- and differentiation (Figs. 4A, B). The numbers of DCX+ human cells did not differ significantly from 2 to 4 weeks (cf. 79.1±6.9% and 74.2±8.4%) indicating a high level of on-going neuronal migration- and differentiation also at 4 weeks (Table 2).

Maturation of neurons into a later developmental state in the co-cultured human cells was shown by 35% more Tau+ human cells, compared to monocultures at 2 weeks, (Table 2). In agreement, complex human Tau+ neuronal morphologies demonstrating a later differentiation state were only found in the co-cultures (Fig. 4F).

Donor-host integration was observed primarily at 4 weeks with DCX/hTau+ cells located in the outer region of the BS tissue (Figs. 4G, H). These cells exhibited profiles of migrating cells or early differentiated neurons.

**Figure 5:**
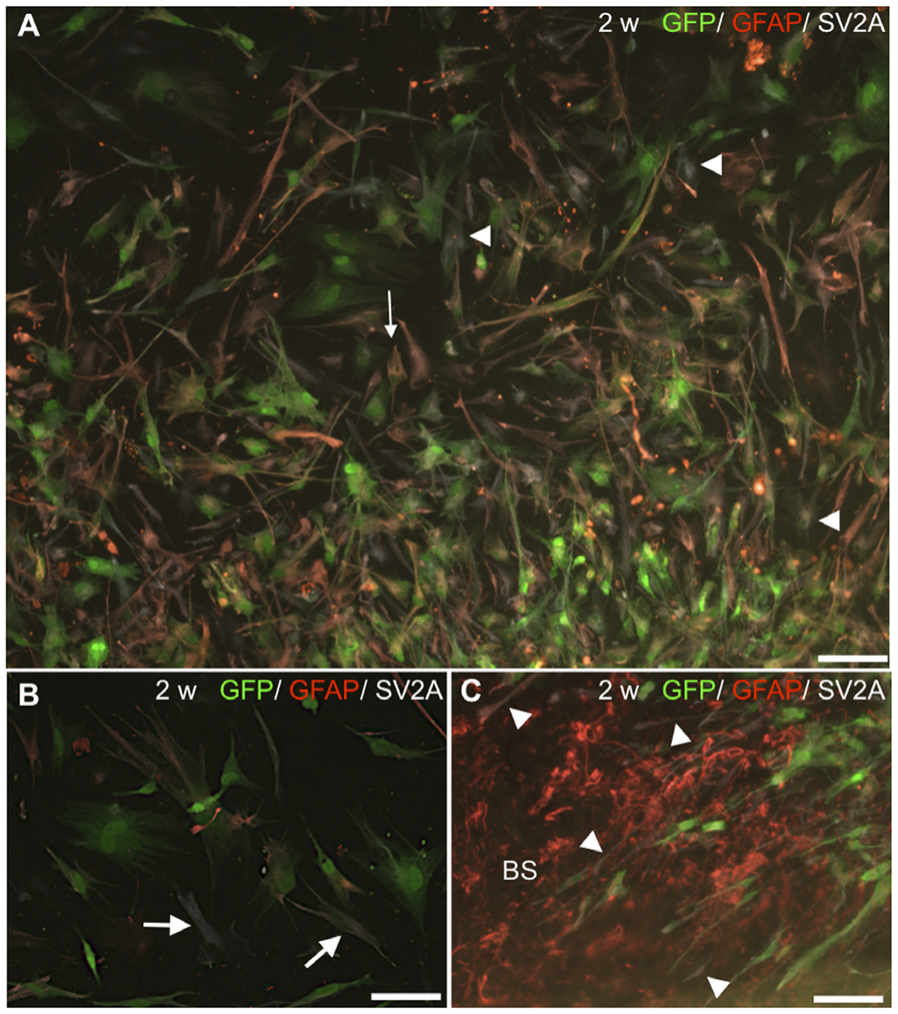
Synapse-formation in co-cultured HNPCs (adjacent deposit). **A**. SV2A (white)- expression pattern was confined largely to the core of the cell mass. SV2A+ HNPC expressing GFAP (red) indicated maturing neuronal progenitors (arrow). It may be suggested that cells with a more mature cellular profile expressing GFP and SV2A but negative for GFAP were of a neuronal phenotype (arrowheads). **B**. High magnification of occasional SV2A (white)/GFP+ HNPCs (arrows). **C**. Notably, GFP/SV2A (white)- expressing processes (arrowheads) indicated a capacity for functional interaction of donor-host cells. BS = brain stem slice. Scale bar equals 100 µm in A and 50 µm in B, C.

Expression of SV2A was found in the HNPCs after co-culture. The SV2A expression pattern was confined largely to the core of the cell mass, staining both immature glial- and neuronal like morphologies (Fig. 5A). SV2A-positive cells had both flat rounded cellular profile and slender cell bodies (Fig. 5A). It can be assumed that cells only positive for SV2A but negative for GFAP were of a neuronal phenotype (Fig. 5B). SV2A-expression was co-localized with GFP+ cells within the host tissue (Fig. 5C). However, in parallel with the SV2A stained monocultures it is difficult to make any conclusions based on this staining as it is contradictory to previous reports on the location of SV2A in the cell [52].

In co-culture at both survival times HNPCs exhibited a later staged neuronal differentiation as compared to monocultures, based on both quantitative data of neuronal marker expression as well as morphological evaluation. In addition, from 2 to 4 weeks of co-culture a clear transition of large numbers of human cells into a neuronal lineage was observed (cf. Figs. 4A, B).

#### HNPCs adjacent to auditory BS slices—glial differentiation

**Figure 6:**
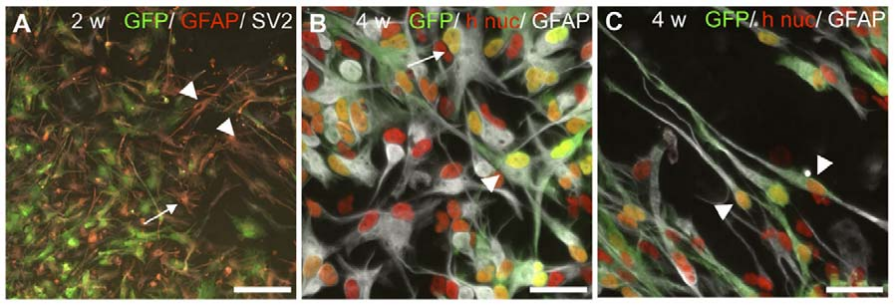
HNPCs differentiate into a glial lineage after co-culture (adjacent deposit). **A**. Typical GFAP+ HNPC morphologies, e.g. with fibroblast- (arrowheads), astrocytic- (arrow) and fusiform- (arrowhead) shaped bodies were found at 2 weeks. **B**. At 4 weeks a minor fraction possessed flat and large cell morphologies were found by GFP/GFAP (arrow), and the majority had elongated cell bodies with long extensive processes (arrowhead). **C**. At 4 weeks GFP/GFAP-expressing cells with morphologies typical of migrating cells were abundantly found. Scale bar equals 100 µm in A; 50 µm in B, C.

A lower number of GFAP+ HNPCs were found after co-culture as compared to monocultures at both time points (Table 2). Co-cultured GFAP-expressing HNPCs were predominantly found at the center but also in the periphery of the sphere (Figs. 6A, B). In the center at 2 weeks the GFAP+ cells mainly displayed immature cell morphologies (Figs. 6A, B). Mature glia was located in the periphery, as judged from their fibroblast-, astrocytic- or fusiform morphologies (Fig. 6A). In addition, GFAP+ processes were extended in length and less branched indicating that these cells were in a migratory stage at 2 weeks (Fig. 6C). At 4 weeks HNPCs with mature glial morphologies outnumbered cells with migratory cell profiles. Moreover, only at 4 weeks, extensive human glial processes were found at the donor cells/host border and reaching into the BS slice. The origin of these processes is not yet clear, but at least a fraction are likely to be human proven by the existence of GFP/human nuclei+ cells within the dense rim at the host tissue border facing the human cell implant (Figs. 3C, G).

In line with the neuronal differentiation for co-cultured HNPCs, the extent of glial differentiation was found in a later phenotypic stage and glial formation continued from 2 to 4 weeks.

#### HNPCs on top of auditory BS slices—survival and migration

**Figure 7:**
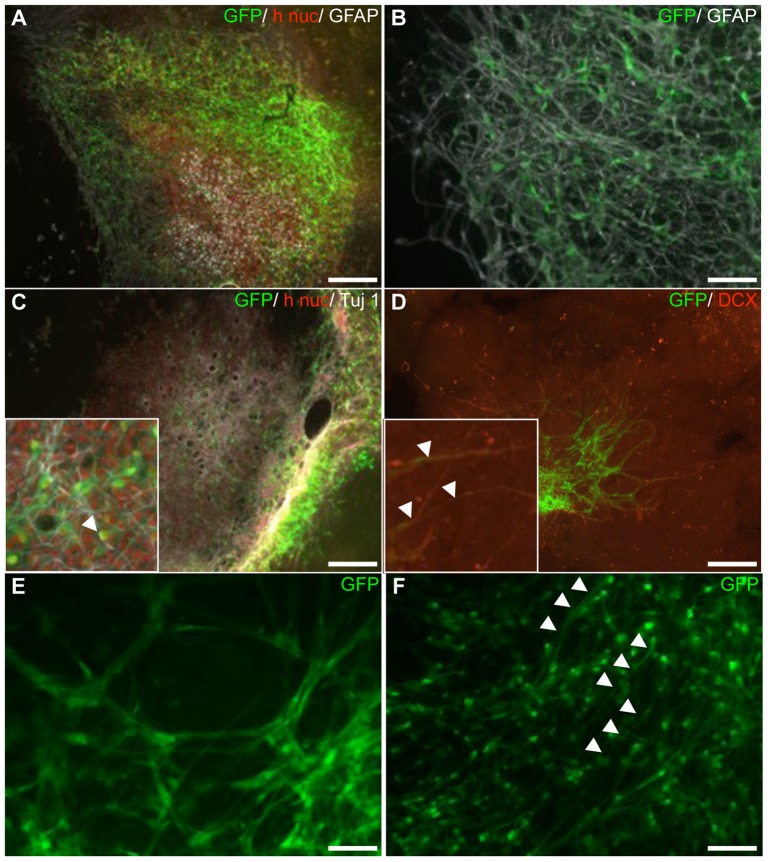
HNPCs cultured on top of BS slices form honeycomb-like networks and primarily neurons. **A**. Distribution of HNPCs detected by h nuc (red)- or GFP (green)- expression was uniform throughout the entire surface of the BS slice. GFAP (white)- expression was mainly seen in the center of the human cell mass. **B**. GFP (green)/GFAP (white)-labelling revealed large numbers of HNPC processes expressing GFAP. **C**. Neuronal differentiation was confirmed by Tuj1 (white)-staining co-localizing with h nuc (red) and GFP (green). Arrowhead in insert shows GFP/Tuj1+ process. **D**. Neuronal differentiation was also confirmed by DCX (red)/GFP+ processes (arrowheads in insert). **E**. A honeycomb-like pattern was formed by GFP+ HNPCs network on the surface of the BS slice. **F**. The vast majority of GFP-labeled cells had morphologies of migrating/differentiating neurons (arrowheads). Scale bar equals 200 µm in A, 100 µm in B; 200 µm in C, D; 50 µm in E, 100 µm in F.

The on-top cultured spheres were included in order to determine if the direct connection of the donor and host was needed to have a permissive effect on survival, migration, phenotypic differentiation as well as integration of HNPC into host tissue. As mentioned above, all spheres deposited on top of the BS slice, in the CN region, survived the co-culture periods (i.e. 2/2 at 2 weeks and 4/4 at 4 weeks, Table 1). The cell survival was proven both by h nuc- and GFP-expressing cells and a large numbers (no quantifiction was made) of cells were found at both survival times. The distribution of HNPCs in these cultures was in a more uniform manner throughout the entire surface of the BS as compared with the co-cultures presented above, but with neither extensive nor directed migration observed (Figs. 7A, C, D). The migration pattern appeared to be confined to the surface of the BS and to some extent deep into the BS slice core without sending large number of processes out from the BS slice. Occasionally, tens of GFP-expressing cells formed an extensive cellular honeycomb-like structure network on the surface of the BS slice, which was never seen in the other two types of cultures (see Fig. 7E).

#### HNPCs on top of auditory BS slices—glial and neuronal differentiation

There was a remarkable difference in the types and proportions of GFP+ cellular profiles found between the two types of co-cultures included, as specified by the placement of the spheres adjacent or on top of the BS slice. Very seldom GFP+ cells cultured on top of the BS-slice were found displaying mature glial morphologies, i.e. fibroblast-, large flat- or astrocytic morphologies (Figs. 7E, F). Instead, the vast majority of the GFP-labeled cells displayed an immature morphology or migrating/differentiating neuronal morphologies (Fig. 7F). A large fraction of GFP+ cells had bipolar profiles, specific for cortical radial glia but also described to be typical of auditory neurons [56].

Extensive GFAP-expression was detected, and these cells primarily exhibited slender bipolar cell bodies with long unbranched processes (Fig. 7B). However, although it was difficult to determine the origin of the GFAP-expressing cells at least some could be identified as human (data not shown). Tuj1-immunoreactivity was co-localized with the GFP marker suggesting neuronal differentiation of human cells (Fig. 7C). Although it was difficult to distinguish Tuj1+ transplanted cells from the host neurons since the former appeared to blend in with the architecture of the slice and spread on its' surface, it was still possible to morphologically distinguish some of Tuj1+ cells with a fusiform-shaped body with polarized elongated processes. Numerous GFP/DCX-labeled cells were found, indicating that many of the transplanted cells were in a transitional stage into more mature phenotypes and were also migrating (Fig. 7D).

Together, HNPCs grown on-top of the BS slice show excellent survival, generate a distribution pattern remarkably different from the two other culture models used (adjacent cultured and monocultured HNPC, respectively) and display neuronal differentiation while no mature glial cells were observed.

## Discussion

### Stem cell therapy for treating sensory-neural hearing loss

The ideal candidate type of cells for a clinical cell replacement therapy aiming at replacing lost SGNs and restoring the AN, is an expandable population of SGN precursors. The previous lack of knowledge concerning the molecular mechanisms on the development of SGNs and, hence, absence of reliable markers for auditory neurons have hindered efforts to convert stem cells into bona fide SGNs. Encouragingly, signals were recently described for the initial specification of auditory neurons and in their progress from axon guidance stages to the on-set of hearing [57], [58].

While awaiting a stable and expandable source of SGN stem cells, we here employed a human neural precursor cell, expandable into high numbers with a maintained ability for multipotent differentiation including neuronal differentiation with long-distance neuronal projections after experimental transplantation [43], [44], [46], [59]. These HNPCs originate from the embryonic forebrain, with the tissue at the time of dissection (9 week) able to generate the neurons and glial cells composing the adult striatum, olfactory bulb and the cortex [45].

Some judge hearing as our most precious and complete sense where partial loss of hearing ability is a severe handicap for the many millions of patients that today suffer from auditory degenerative diseases [1]. Current available hearing aids, such as cochlear implants, need functional SGNs in order to offer full hearing. Thus, patients with auditory nerve atrophy will not fully benefit from cochlear implants [60]. Stem cell transplantation in neurodegenerative disorders has been intensively studied over the last two decades. In agreement, reports by others and us support the development of a cellular replacement strategy for auditory deficiencies including neuronal loss [14]–[19], [21], [22], [33], [40]–[42], [61]. Results are emerging both on functional benefits after grafting to disease models as well as demonstration of a protocol for induction of auditory neurons from human embryonic stem cells (hES) [32]. Lately, grafted hES-derived auditory neurons were reported to significantly improve auditory-evoked response thresholds to a model of toxin (ouabain)-induced deafness [58].

In line with reports on other cultured CNS regions our protocol for culturing organotypic cultures of the post-natal rodent brainstem demonstrates a preserved 3D cytoarchitecture for up to five weeks resembling the corresponding region *in vivo*, which offers a unique ability to test the potential of donor cells to integrate with appropriate parts of the auditory neural circuit [40], [41], [62]–[67]. In our previous co-culture studies the properties of mouse ESCs were efficiently explored [40], [41].

### HNPCs survive well after co-culture with auditory BS slices

A requirement of a candidate donor cell is good long-term survival within the host tissue. We reported excellent survival up to over a year of the present used human cells after grafting to various regions of the developing and adult rodent brain [43], [44]. In addition, these HNPCs have been extensively and repeatedly used as an *in vitro* cell-based assay for testing differentiation protocols [45], [59] and their neuronal rescuing effect when co-cultured with explanted retina [68].

In this study, excellent survival (100%) was demonstrated for all co-cultured spheres. In contrast, monocultured spheres had a 58% survival rate at 2 weeks, whereas only 22% of the seeded spheres had survived at 4 w. Moreover, the fraction of surviving HNPCs in the deposited spheres was very low in some of the monocultures (<50 cells) as compared to the abundant numbers of HNPCs found after co-culture. Notably, both during expansion and differentiation *in vitro* the currently used human cell line is dependent on a certain cell density in the culture for survival (non-published data). The low number of surviving monocultured HNPCs limited the ability to quantify an appropriate number of specimens for the comparison of phenotypic differentiation to co-cultured human cells. In agreement, we showed that mouse ESCs co-cultured with the BS slice survive significantly better than monocultured ECSs [40], [41].

Our findings demonstrate that HNPCs survive well in the presence of an explanted BS slice, which is prerequisite for further investigations on improvement of donor-host integration. The better survival of both co-cultured HNPCs and mouse ESCs after co-culture, suggest that the BS slice culture exert factors that are permissive for survival.

### HNPCs migrate towards and to some extent into the auditory BS slices

HNPCs demonstrated a capacity to migrate and distribute both after mono- and co-culture. In both culture systems a uniform migration was found with scattered cells located at short distance from the remaining HNPC cell mass. However, migration of human cells in an ordered fashion was only observed in co-cultures and always directed toward the BS slice. These cells often formed chains of migrating bipolar cells, expressing either GFAP or neuronal markers (Tuj1, DCX, hTau). The HNPCs responding to eventual attractant cues for migration exerted by the BS slice may be the population present within the cell line destined to migrate in a specific mode, such as cortical radial glial cells, cortical neuronal progenitors and olfactory bulb neuronal progenitors [45], [69]. In parallel, mouse ESCs displayed a similar migration pattern, including directed migration of neuronal progenitors toward the BS slice [40], [41]. We as well as others have shown the possibility for other types of stem cells to migrate on the surface of and into the host tissue in similar organotypic culture systems [62]–[67] in the cultures where the spheres were placed on top of the BS slice migration was confined to the BS surface only, indicating absence/low impact of intrinsic and/or extrinsic cues guiding cell migration out from the BS tissue.

Quantification of the area covered with human cells, as a measurement of distribution/migration ability, showed no statistical difference between mono- and co-cultured cells. This can probably be explained by the large variance within the respective groups, which could depend on the difficulties to select sphere of precisely sized 0.3 and 1.0 mm, respectively. But, notably, for the specimens including use of 1 mm large spheres the distribution area was about 36% and 46% larger for co-cultured HNPCs, at 2 and 4 weeks. This finding indicates that the conditioned microenvironment in the co-culture is more permissive for a certain population of HNPCs.

Previous *in vivo* intracerebral transplantation studies using healthy rodents, with the current used HNPCs describe long distance-migration (tens of millimeters) in both grey and white matter, target directed migration in neurogenic regions as well as non-directed migration in non-neurogenic regions further indicating the ability of these HNPCs to respond to extrinsic cues in different neural microenvironments [43], [44], [69].

Integration of the HNPCs with host tissue was observed, but to a limited extent. The sparse integration of donor cells into host tissue may be explained by several factors: i) the time given for integration to occur was too short, ii) the presence of a glial cellular border of host or donor origin blocking the migration/neurite extensions, iii) the human cells had difficulties in penetrating the extracellular matrix of the BS, iv) a lack of cues guiding the cells into the BS tissue could be explained by the *in vitro* situation, v) the cells did not possess the intrinsic capacity to migrate longer distances under the given circumstances or vi) it was simply not possible to detect the human cells that had actually migrated into the BS tissue. The last argument might be due to down-regulation of the GFP reporter gene [43], [44], [46] and the human nuclei marker used here [43], [44], [70] upon differentiation of the cells into more mature phenotypes. In further experiments exploring the full capacity of the human cells to integrate with host cells additional human-specific markers can be added, such as a marker for human mitochondria [43], [44]. In previous studies, results are contradictory on the capacity of similar expanded HNPC to migrate and integrate with host tissue after grafting to models of neurodegeneration, see e.g. [10], [71].

Together, the ability of the HNPCs to actually migrate close to and to some extent send neurite processes toward/within host tissue encourages further studies of transplantation of these cells into the corresponding *in vivo* environment. Secondly, the reproducible HNPCs/BS slice culture offers an excellent controlled model for testing pharmacological treatments for improving donor/host integration.

### Auditory BS slice culture exerts factors permissive for phenotypic HNPC differentiation

The most important finding here is the increased phenotypic differentiation demonstrated in the co-cultured HNPCs compared to monocultures. Firstly, the substantial lower number (about 20%) of nestin+ co-cultured HNPCs as compared to controls found at 2 weeks indicate that the BS slice exert differentiation-promoting factors. Secondly, a related significant decrease in the number (about 20%) of GFAP+ cells (expressed in neural progenitor cells) in co-cultured HNPCs from 2 to 4 week of culture suggests a shift of a major fraction of immature cells into a more mature phenotypic stage. In agreement, both the fraction of nestin+ and GFAP+ HNPCs in monocultures was similar at 2 and 4 weeks.

The analysis was focused on the extent of neuronal differentiation capacity and integration with host tissue of the HNPCs. The currently demonstrated results are in line with our previous studies illustrating a significantly higher number of mouse ESCs generating neuronal phenotypes in co-culture vs monoculture [40], [41]. Here the combined analysis of neuronal marker expression and detailed morphological analysis imply that the co-culture is permissive for overall neuronal cell development, including neuroblast migration and development into later phenotypic stages.

A later staged neuronal maturation was demonstrated in the co-cultured HNPCs compared to monocultures. In co-cultures immunohistochemical analysis i) the number of Tuj1+HNPC was higher (17%) at the longer survival time, indicating a continuously increasing neuronal population, ii) already at 2 weeks 35% more co-cultured HNPCs expressed the mature neuronal marker Tau than monocultures and iii) hTau+ cells decreased over time for the co-cultures, which could be explained by a translocation of this microtubule associated protein to the processes of the cells making them difficult to include in the cell countings.

Morphological analysis of all specimens revealed no monocultured HNPCs with migrating/mature neuronal cellular profiles. In contrast, a large proportion of the co-cultured HNPCs displayed morphologies of neurons ranging from migrating neuroblasts to multipolar later stage neuronal profiles. The neuronal morphologies were accompanied by the expression of one to several of the neuronal markers included.

Last, “neurogenic HNPC clusters”, judged as early maturing/proliferating neuronal progenitors, were only found in the co-cultures. In addition, a gradient of increasing numbers of neuronal cells among the HNPC population was noted closer to the BS slice. In co-cultures with adjacently placed spheres a remarkably higher number of DCX-positive cells were located close to the BS slice as well as at the periphery of the entire distribution area of the HNPC. In agreement, the same expression pattern was also found for the neuronal markers, Tuj1 and Tau. Taken together, this indicates that an established gradient of cues affect HNPC-migration and neuronal formation.

The distance to host tissue clearly affected the behavior of the co-cultured HNPCs, with much less glial differentiation observed for the on top deposited HNPCs as compared to when the spheres were placed adjacent to the BS slice. Taken together the results from the two different co-culture approaches indicate that the HNPCs are highly capable of responding to small changes in extrinsic signals for phenotypic differentiation.

No functional analysis of the HNPC-derived neurons was included in the present study based on the small numbers of co-cultured HNPCs with mature complex neuronal morphologies and donor-derived neurites found, respectively. If improvement on HNPC-derived neural integration with the auditory circuitry can be achieved it is of great interest to examine the extent of functional potential of the donor cells by studying e.g. neurotransmitter release (glutamate [72]) and electrophysiological properties [58].

### Concluding remarks

Here we initially describe the use of human neural precursor cells as a tool for exploring the key events for a successful future clinical cell based therapy for treating deafness. Although the exact stem cells source may not be the same in a clinical setting, the neural progeny generated here will most likely be a similar type during the process of integration with the neural host tissue of interest. In further studies we will elucidate the full potential of these HNPCs to integrate with the BS slice by manipulating the host glial response and after transplantation to a model of toxin-induced deafness in rodents. Two other requirements for a candidate donor cell, apart from morphological integration, which require exploration are functional integration studied by using e.g. electrophysiological cell recordings and release of appropriate neurotransmitters, i.e. glutamate. In conclusion, the encouraging results presented here with regard to the excellent survival, late stage neuronal differentiation and directed migration of the human neural cells after co-culture with the BS slice model inspire us to further investigate the ability to develop a cell based therapy for treating hearing loss.
